# Bis(1*H*-benzotriazole-7-sulfonato-κ*O*)bis­(1,10-phenanthroline-κ^2^ *N*,*N*′)cadmium dihydrate

**DOI:** 10.1107/S1600536811045971

**Published:** 2011-11-05

**Authors:** Xiao-Hong Zhu, Xiao-Chun Cheng

**Affiliations:** a Faculty of Life Science and Chemical Engineering, Huaiyin Institute of Technology, Huaian 223003, People’s Republic of China

## Abstract

In the title complex, [Cd(C_6_H_4_N_3_O_3_S)_2_(C_12_H_8_N_2_)_2_]·2H_2_O, the Cd^2+^ cation is located on an inversion center and is coordinated by four N atoms from two symmetry-related 1,10-phenanthroline ligands and two sulfonate O atoms from two benzotriazole-7-sulfonate anions, displaying a distorted CdN_4_O_2_ octa­hedral geometry. In the crystal, O—H⋯N, O—H⋯O, N—H⋯O, C—H⋯N and C—H⋯O hydrogen bonds occur. The lattice water mol­ecules and sulfonate O atoms as donor or acceptor atoms play important roles in the formation of these inter­actions.

## Related literature

For related structures, see: Xia *et al.* (2010 ▶).
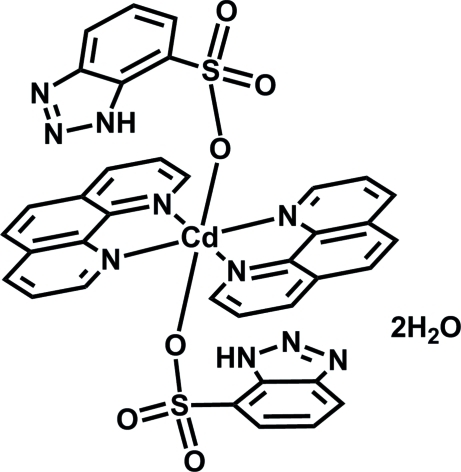


## Experimental

### 

#### Crystal data

[Cd(C_6_H_4_N_3_O_3_S)_2_(C_12_H_8_N_2_)_2_]·2H_2_O
*M* *_r_* = 905.20
Triclinic, 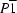

*a* = 7.5675 (16) Å
*b* = 10.238 (2) Å
*c* = 11.974 (2) Å
α = 79.852 (2)°
β = 77.948 (3)°
γ = 84.092 (3)°
*V* = 891.0 (3) Å^3^
*Z* = 1
Mo *K*α radiation
μ = 0.80 mm^−1^
*T* = 293 K
0.20 × 0.12 × 0.12 mm

#### Data collection

Bruker SMART APEXII CCD diffractometer
Absorption correction: multi-scan (*SADABS*; Sheldrick, 1996 ▶) *T*_min_ = 0.856, *T*_max_ = 0.910
4887 measured reflections
3431 independent reflections
3174 reflections with *I* > 2σ(*I*)
*R*_int_ = 0.017

#### Refinement

*R*[*F* ^2^ > 2σ(*F* ^2^)] = 0.038
*wR*(*F* ^2^) = 0.092
*S* = 1.06
3431 reflections
235 parameters
H-atom parameters constrained
Δρ_max_ = 0.51 e Å^−3^
Δρ_min_ = −0.79 e Å^−3^

### 

Data collection: *APEX2* (Bruker, 2008 ▶); cell refinement: *SAINT* (Bruker, 2008 ▶); data reduction: *SAINT*; program(s) used to solve structure: *SHELXS97* (Sheldrick, 2008 ▶); program(s) used to refine structure: *SHELXL97* (Sheldrick, 2008 ▶); molecular graphics: *DIAMOND* (Brandenburg, 2000 ▶); software used to prepare material for publication: *SHELXTL* (Sheldrick, 2008 ▶).

## Supplementary Material

Crystal structure: contains datablock(s) I, global. DOI: 10.1107/S1600536811045971/pv2475sup1.cif

Structure factors: contains datablock(s) I. DOI: 10.1107/S1600536811045971/pv2475Isup2.hkl

Supplementary material file. DOI: 10.1107/S1600536811045971/pv2475Isup3.cdx

Additional supplementary materials:  crystallographic information; 3D view; checkCIF report

## Figures and Tables

**Table 1:** Hydrogen-bond geometry (Å, °)

*D*—H⋯*A*	*D*—H	H⋯*A*	*D*⋯*A*	*D*—H⋯*A*
O1*W*—H1*WA*⋯N5^i^	0.98	2.10	3.015 (4)	155
O1*W*—H1*W*⋯O3^ii^	0.98	2.00	2.934 (5)	158
N3—H3*N*⋯O1^iii^	0.90	2.21	3.009 (4)	148
C6—H6⋯O2	0.93	2.60	2.947 (4)	103
C8—H8⋯N4^iv^	0.93	2.42	3.306 (6)	159
C14—H14⋯O1*W*	0.93	2.51	3.414 (5)	164

Symmetry codes: (i) 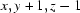; (ii) 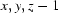; (iii) 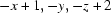; (iv) 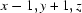.

**Fig. 1.**
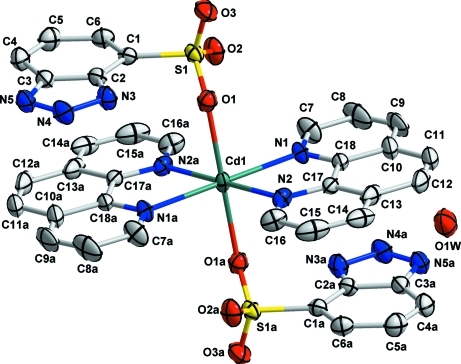
The coordination environment of Cd ion in the title complex with the ellipsoids drawn at the 30% probability level. The hydrogen atoms have been omitted for clarity. Symmetry code: a = -x, -y, -z+2.

## supplementary crystallographic information

### Comment

Benzotriazole-7-sulfonic acid are often used as ligand to synthesize complexes for its variable coordination modes. Herein, we report the crystal structure of title complex. The asymmetric unit consists of half of one cadmium ion, one 1,10-phenanthroline molecule, one benzotriazole-7-sulfonate anion, and one lattice water molecule. The Cd ion is located on an inversion center and coordinated by four N atoms from two different 1,10-phenanthroline molecules and two sulfonate O atoms from two different benzotriazole-7-sulfonate anions, displaying a distorted CdN_4_O_2_ octahedral geometry (Fig. 1). Benzotriazole-7-sulfonate shows a monodentate coordinating mode, while 1,10-phenanthroline displays a bidentate chelating coordinating mode. In the crystal structure, there exist O—H···N, O—H···O, N—H···O, C—H···N, and C—H···O hydrogen bonds (Table 1). Lattice water molecules and sulfonate O atoms as donor or acceptor play very important roles in the formation of these hydrogen bonding interactions.

### Experimental

A mixture of cadmium perchlorate hexahydrate (83.9 mg, 0.2 mmol), benzotriazole-7-sulfonic acid (39.8 mg, 0.2 mmol), 1,10-phenanthroline (36.0 mg, 0.2 mmol) and potassium hydroxide (11.2 mg, 0.2 mmol) in 12 ml H_2_O was sealed in a 16 ml Teflon-lined stainless steel container and heated to 393 K for 3 days. After cooling to room temperature, colorless block crystals of the title complex were obtained.

### Refinement

The hydrogen atoms bonded to C atoms were located in geometrically idealized positions and constrained to ride on their parent atoms, with C—H = 0.93 Å and *U*_iso_(H) = 1.2*U*_eq_(C). The hydrogen atoms bonded to N3 and O1W were found from a difference Fourier map and fixed at those position with *U*_iso_(H) = 1.2*U*_eq_(N or O).

### Crystal data

**Table:**

[Cd(C_6_H_4_N_3_O_3_S)_2_(C_12_H_8_N_2_)_2_]·2H_2_O	*Z* = 1
*M**_r_* = 905.20	*F*(000) = 458
Triclinic, *P*1	*D*_x_ = 1.687 Mg m^−^^3^
Hall symbol: -P 1	Mo *K*α radiation, λ = 0.71073 Å
*a* = 7.5675 (16) Å	Cell parameters from 2099 reflections
*b* = 10.238 (2) Å	θ = 2.5–25.3°
*c* = 11.974 (2) Å	µ = 0.80 mm^−^^1^
α = 79.852 (2)°	*T* = 293 K
β = 77.948 (3)°	Block, colorless
γ = 84.092 (3)°	0.20 × 0.12 × 0.12 mm
*V* = 891.0 (3) Å^3^	

### Data collection

**Table:**

Bruker SMART APEXII CCD diffractometer	3431 independent reflections
Radiation source: fine-focus sealed tube	3174 reflections with *I* > 2σ(*I*)
graphite	*R*_int_ = 0.017
phi and ω scans	θ_max_ = 26.0°, θ_min_ = 2.0°
Absorption correction: multi-scan (*SADABS*; Sheldrick, 1996)	*h* = −9→9
*T*_min_ = 0.856, *T*_max_ = 0.910	*k* = −12→9
4887 measured reflections	*l* = −14→14

### Refinement

**Table:**

Refinement on *F*^2^	Primary atom site location: structure-invariant direct methods
Least-squares matrix: full	Secondary atom site location: difference Fourier map
*R*[*F*^2^ > 2σ(*F*^2^)] = 0.038	Hydrogen site location: inferred from neighbouring sites
*wR*(*F*^2^) = 0.092	H-atom parameters constrained
*S* = 1.06	*w* = 1/[σ^2^(*F*_o_^2^) + (0.0459*P*)^2^ + 0.3547*P*] where *P* = (*F*_o_^2^ + 2*F*_c_^2^)/3
3431 reflections	(Δ/σ)_max_ < 0.001
235 parameters	Δρ_max_ = 0.51 e Å^−^^3^
0 restraints	Δρ_min_ = −0.79 e Å^−^^3^

### Special details

**Table:**

Geometry. All e.s.d.'s (except the e.s.d. in the dihedral angle between two l.s. planes) are estimated using the full covariance matrix. The cell e.s.d.'s are taken into account individually in the estimation of e.s.d.'s in distances, angles and torsion angles; correlations between e.s.d.'s in cell parameters are only used when they are defined by crystal symmetry. An approximate (isotropic) treatment of cell e.s.d.'s is used for estimating e.s.d.'s involving l.s. planes.
Refinement. Refinement of *F*^2^ against ALL reflections. The weighted *R*-factor *wR* and goodness of fit *S* are based on *F*^2^, conventional *R*-factors *R* are based on *F*, with *F* set to zero for negative *F*^2^. The threshold expression of *F*^2^ > σ(*F*^2^) is used only for calculating *R*-factors(gt) *etc*. and is not relevant to the choice of reflections for refinement. *R*-factors based on *F*^2^ are statistically about twice as large as those based on *F*, and *R*- factors based on ALL data will be even larger.

### Fractional atomic coordinates and isotropic or equivalent isotropic displacement parameters (Å^2^)

**Table:**

	*x*	*y*	*z*	*U*_iso_*/*U*_eq_	
C1	0.2876 (4)	−0.0551 (3)	1.2678 (3)	0.0391 (7)	
C2	0.3840 (4)	−0.1707 (3)	1.2340 (3)	0.0411 (7)	
C3	0.3952 (5)	−0.2877 (3)	1.3118 (3)	0.0486 (8)	
C4	0.3135 (6)	−0.2931 (4)	1.4286 (3)	0.0617 (10)	
H4	0.3237	−0.3703	1.4816	0.074*	
C5	0.2185 (6)	−0.1815 (4)	1.4619 (3)	0.0616 (10)	
H5	0.1615	−0.1830	1.5388	0.074*	
C6	0.2047 (5)	−0.0634 (4)	1.3821 (3)	0.0503 (8)	
H6	0.1377	0.0107	1.4077	0.060*	
C7	−0.1788 (6)	0.2998 (4)	1.0384 (4)	0.0667 (11)	
H7	−0.2190	0.2593	1.1141	0.080*	
C8	−0.2256 (7)	0.4350 (4)	1.0076 (6)	0.0888 (16)	
H8	−0.2956	0.4828	1.0625	0.107*	
C9	−0.1697 (8)	0.4960 (4)	0.8992 (6)	0.094	
H9	−0.2012	0.5858	0.8782	0.113*	
C10	−0.0633 (7)	0.4231 (4)	0.8176 (5)	0.0826 (11)	
C11	0.0040 (7)	0.4799 (4)	0.7000 (5)	0.0826 (11)	
H11	−0.0223	0.5698	0.6752	0.099*	
C12	0.1031 (7)	0.4063 (5)	0.6261 (4)	0.086	
H12	0.1431	0.4461	0.5501	0.103*	
C13	0.1509 (6)	0.2687 (5)	0.6583 (3)	0.0669 (12)	
C14	0.2591 (6)	0.1892 (5)	0.5828 (3)	0.075	
H14	0.3036	0.2258	0.5065	0.090*	
C15	0.2986 (6)	0.0608 (6)	0.6201 (3)	0.0778 (15)	
H15	0.3700	0.0080	0.5699	0.093*	
C16	0.2322 (5)	0.0062 (4)	0.7349 (3)	0.0553 (9)	
H16	0.2599	−0.0835	0.7596	0.066*	
C17	0.0889 (5)	0.2084 (3)	0.7731 (3)	0.0442 (8)	
C18	−0.0206 (5)	0.2869 (3)	0.8538 (3)	0.0446 (8)	
Cd1	0.0000	0.0000	1.0000	0.03563 (12)	
N1	−0.0792 (4)	0.2266 (2)	0.9638 (2)	0.0440 (6)	
N2	0.1306 (4)	0.0781 (3)	0.8100 (2)	0.0412 (6)	
N3	0.4811 (4)	−0.2013 (3)	1.1326 (3)	0.0528 (7)	
H3N	0.5145	−0.1463	1.0661	0.063*	
N4	0.5466 (5)	−0.3294 (3)	1.1468 (3)	0.0649 (9)	
N5	0.4973 (5)	−0.3827 (3)	1.2536 (3)	0.0609 (8)	
O1	0.2630 (3)	0.0480 (2)	1.06094 (19)	0.0507 (6)	
O2	0.1125 (4)	0.1690 (2)	1.2157 (2)	0.0639 (7)	
O3	0.4397 (4)	0.1563 (3)	1.1556 (3)	0.0694 (8)	
O1W	0.5025 (6)	0.3191 (3)	0.3203 (3)	0.1043 (13)	
H1W	0.4536	0.2802	0.2647	0.125*	
H1WA	0.5382	0.4094	0.2870	0.125*	
S1	0.27401 (12)	0.09358 (8)	1.16896 (7)	0.0434 (2)	

### Atomic displacement parameters (Å^2^)

**Table:**

	*U*^11^	*U*^22^	*U*^33^	*U*^12^	*U*^13^	*U*^23^
C1	0.0376 (18)	0.0377 (16)	0.0447 (17)	−0.0022 (13)	−0.0102 (14)	−0.0116 (13)
C2	0.0438 (19)	0.0382 (17)	0.0441 (17)	−0.0036 (14)	−0.0117 (14)	−0.0097 (14)
C3	0.049 (2)	0.0364 (17)	0.063 (2)	−0.0067 (15)	−0.0189 (17)	−0.0031 (15)
C4	0.070 (3)	0.056 (2)	0.057 (2)	−0.017 (2)	−0.016 (2)	0.0095 (18)
C5	0.064 (3)	0.073 (3)	0.045 (2)	−0.014 (2)	−0.0032 (18)	−0.0050 (18)
C6	0.051 (2)	0.054 (2)	0.0463 (19)	−0.0015 (17)	−0.0062 (16)	−0.0147 (16)
C7	0.065 (3)	0.048 (2)	0.084 (3)	0.0110 (19)	0.000 (2)	−0.027 (2)
C8	0.075 (3)	0.045 (2)	0.154 (5)	0.021 (2)	−0.028 (3)	−0.045 (3)
C9	0.103	0.027	0.169	0.013	−0.073	−0.019
C10	0.104 (3)	0.0401 (15)	0.112 (3)	−0.0210 (16)	−0.064 (2)	0.0255 (15)
C11	0.104 (3)	0.0401 (15)	0.112 (3)	−0.0210 (16)	−0.064 (2)	0.0255 (15)
C12	0.108	0.079	0.073	−0.052	−0.049	0.044
C13	0.076 (3)	0.086 (3)	0.0399 (19)	−0.044 (2)	−0.0207 (19)	0.0185 (19)
C14	0.081	0.116	0.033	−0.067	−0.006	0.005
C15	0.055 (3)	0.140 (5)	0.044 (2)	−0.036 (3)	0.0090 (18)	−0.033 (2)
C16	0.043 (2)	0.078 (3)	0.0448 (19)	−0.0017 (18)	0.0003 (15)	−0.0201 (18)
C17	0.048 (2)	0.0476 (19)	0.0391 (17)	−0.0175 (15)	−0.0180 (15)	0.0062 (14)
C18	0.049 (2)	0.0306 (16)	0.058 (2)	−0.0073 (14)	−0.0262 (16)	0.0029 (14)
Cd1	0.0452 (2)	0.02687 (17)	0.03025 (17)	0.00340 (13)	−0.00184 (13)	−0.00229 (11)
N1	0.0470 (17)	0.0291 (13)	0.0531 (16)	0.0042 (12)	−0.0065 (13)	−0.0072 (12)
N2	0.0385 (15)	0.0498 (16)	0.0335 (13)	−0.0028 (12)	−0.0031 (11)	−0.0065 (11)
N3	0.062 (2)	0.0408 (16)	0.0515 (17)	0.0086 (14)	−0.0040 (14)	−0.0127 (13)
N4	0.075 (2)	0.0414 (17)	0.081 (2)	0.0146 (16)	−0.0183 (19)	−0.0232 (17)
N5	0.069 (2)	0.0371 (16)	0.081 (2)	0.0011 (15)	−0.0253 (18)	−0.0088 (16)
O1	0.0553 (15)	0.0516 (14)	0.0439 (13)	0.0002 (11)	−0.0099 (11)	−0.0066 (10)
O2	0.0668 (18)	0.0477 (14)	0.0699 (17)	0.0178 (13)	−0.0034 (14)	−0.0144 (12)
O3	0.0634 (18)	0.0540 (16)	0.093 (2)	−0.0199 (13)	−0.0242 (15)	0.0015 (14)
O1W	0.182 (4)	0.066 (2)	0.066 (2)	−0.025 (2)	−0.032 (2)	0.0030 (16)
S1	0.0464 (5)	0.0329 (4)	0.0505 (5)	0.0015 (3)	−0.0090 (4)	−0.0088 (3)

### Geometric parameters (Å, °)

**Table:**

C1—C6	1.373 (5)	C13—C14	1.402 (7)
C1—C2	1.402 (4)	C13—C17	1.408 (5)
C1—S1	1.764 (3)	C14—C15	1.336 (7)
C2—N3	1.351 (4)	C14—H14	0.9300
C2—C3	1.388 (4)	C15—C16	1.397 (5)
C3—N5	1.375 (5)	C15—H15	0.9300
C3—C4	1.400 (5)	C16—N2	1.330 (4)
C4—C5	1.361 (6)	C16—H16	0.9300
C4—H4	0.9300	C17—N2	1.356 (4)
C5—C6	1.412 (5)	C17—C18	1.438 (5)
C5—H5	0.9300	C18—N1	1.356 (4)
C6—H6	0.9300	Cd1—N2^i^	2.319 (2)
C7—N1	1.329 (4)	Cd1—N2	2.319 (2)
C7—C8	1.398 (6)	Cd1—N1	2.323 (2)
C7—H7	0.9300	Cd1—N1^i^	2.323 (2)
C8—C9	1.339 (8)	Cd1—O1	2.381 (2)
C8—H8	0.9300	Cd1—O1^i^	2.381 (2)
C9—C10	1.401 (8)	N3—N4	1.349 (4)
C9—H9	0.9300	N3—H3N	0.8963
C10—C18	1.410 (5)	N4—N5	1.291 (5)
C10—C11	1.434 (7)	O1—S1	1.471 (2)
C11—C12	1.325 (7)	O2—S1	1.441 (3)
C11—H11	0.9300	O3—S1	1.434 (3)
C12—C13	1.423 (7)	O1W—H1W	0.9832
C12—H12	0.9300	O1W—H1WA	0.9813
			
C6—C1—C2	116.5 (3)	C16—C15—H15	120.1
C6—C1—S1	121.7 (3)	N2—C16—C15	122.2 (4)
C2—C1—S1	121.8 (2)	N2—C16—H16	118.9
N3—C2—C3	104.3 (3)	C15—C16—H16	118.9
N3—C2—C1	133.9 (3)	N2—C17—C13	121.7 (4)
C3—C2—C1	121.8 (3)	N2—C17—C18	119.0 (3)
N5—C3—C2	108.4 (3)	C13—C17—C18	119.3 (3)
N5—C3—C4	130.9 (3)	N1—C18—C10	121.9 (4)
C2—C3—C4	120.7 (3)	N1—C18—C17	118.2 (3)
C5—C4—C3	117.7 (3)	C10—C18—C17	119.9 (4)
C5—C4—H4	121.1	N2^i^—Cd1—N2	180.0
C3—C4—H4	121.1	N2^i^—Cd1—N1	107.73 (9)
C4—C5—C6	121.4 (4)	N2—Cd1—N1	72.27 (9)
C4—C5—H5	119.3	N2^i^—Cd1—N1^i^	72.27 (9)
C6—C5—H5	119.3	N2—Cd1—N1^i^	107.73 (9)
C1—C6—C5	121.8 (3)	N1—Cd1—N1^i^	180.000 (1)
C1—C6—H6	119.1	N2^i^—Cd1—O1	90.25 (8)
C5—C6—H6	119.1	N2—Cd1—O1	89.75 (9)
N1—C7—C8	122.7 (4)	N1—Cd1—O1	89.33 (9)
N1—C7—H7	118.7	N1^i^—Cd1—O1	90.67 (9)
C8—C7—H7	118.7	N2^i^—Cd1—O1^i^	89.75 (9)
C9—C8—C7	120.0 (4)	N2—Cd1—O1^i^	90.25 (8)
C9—C8—H8	120.0	N1—Cd1—O1^i^	90.67 (9)
C7—C8—H8	120.0	N1^i^—Cd1—O1^i^	89.33 (9)
C8—C9—C10	119.4 (4)	O1—Cd1—O1^i^	180.0
C8—C9—H9	120.3	C7—N1—C18	118.1 (3)
C10—C9—H9	120.3	C7—N1—Cd1	126.6 (3)
C9—C10—C18	118.0 (4)	C18—N1—Cd1	115.3 (2)
C9—C10—C11	123.4 (4)	C16—N2—C17	118.6 (3)
C18—C10—C11	118.7 (5)	C16—N2—Cd1	126.2 (2)
C12—C11—C10	120.9 (4)	C17—N2—Cd1	115.0 (2)
C12—C11—H11	119.5	N4—N3—C2	110.1 (3)
C10—C11—H11	119.5	N4—N3—H3N	121.2
C11—C12—C13	122.7 (4)	C2—N3—H3N	128.2
C11—C12—H12	118.7	N5—N4—N3	109.4 (3)
C13—C12—H12	118.7	N4—N5—C3	107.9 (3)
C14—C13—C17	117.6 (4)	S1—O1—Cd1	128.09 (14)
C14—C13—C12	123.9 (4)	H1W—O1W—H1WA	110.4
C17—C13—C12	118.5 (4)	O3—S1—O2	115.18 (18)
C15—C14—C13	120.1 (3)	O3—S1—O1	110.29 (17)
C15—C14—H14	119.9	O2—S1—O1	113.12 (16)
C13—C14—H14	119.9	O3—S1—C1	107.12 (15)
C14—C15—C16	119.8 (4)	O2—S1—C1	106.42 (16)
C14—C15—H15	120.1	O1—S1—C1	103.81 (14)
			
C6—C1—C2—N3	−179.8 (4)	N2—Cd1—N1—C7	−179.0 (3)
S1—C1—C2—N3	−0.1 (5)	N1^i^—Cd1—N1—C7	−167 (100)
C6—C1—C2—C3	0.1 (5)	O1—Cd1—N1—C7	−89.0 (3)
S1—C1—C2—C3	179.8 (3)	O1^i^—Cd1—N1—C7	91.0 (3)
N3—C2—C3—N5	−0.3 (4)	N2^i^—Cd1—N1—C18	−176.9 (2)
C1—C2—C3—N5	179.8 (3)	N2—Cd1—N1—C18	3.1 (2)
N3—C2—C3—C4	178.2 (3)	N1^i^—Cd1—N1—C18	16 (100)
C1—C2—C3—C4	−1.7 (5)	O1—Cd1—N1—C18	93.1 (2)
N5—C3—C4—C5	−179.8 (4)	O1^i^—Cd1—N1—C18	−86.9 (2)
C2—C3—C4—C5	2.1 (6)	C15—C16—N2—C17	0.7 (5)
C3—C4—C5—C6	−0.9 (6)	C15—C16—N2—Cd1	177.0 (3)
C2—C1—C6—C5	1.1 (5)	C13—C17—N2—C16	−0.2 (5)
S1—C1—C6—C5	−178.6 (3)	C18—C17—N2—C16	−179.9 (3)
C4—C5—C6—C1	−0.7 (6)	C13—C17—N2—Cd1	−176.9 (3)
N1—C7—C8—C9	0.0 (7)	C18—C17—N2—Cd1	3.4 (4)
C7—C8—C9—C10	−0.4 (8)	N2^i^—Cd1—N2—C16	48 (100)
C8—C9—C10—C18	0.5 (7)	N1—Cd1—N2—C16	−179.8 (3)
C8—C9—C10—C11	−179.4 (5)	N1^i^—Cd1—N2—C16	0.2 (3)
C9—C10—C11—C12	−179.5 (5)	O1—Cd1—N2—C16	90.8 (3)
C18—C10—C11—C12	0.6 (7)	O1^i^—Cd1—N2—C16	−89.2 (3)
C10—C11—C12—C13	−0.9 (7)	N2^i^—Cd1—N2—C17	−135 (100)
C11—C12—C13—C14	−178.4 (4)	N1—Cd1—N2—C17	−3.4 (2)
C11—C12—C13—C17	0.5 (6)	N1^i^—Cd1—N2—C17	176.6 (2)
C17—C13—C14—C15	0.7 (6)	O1—Cd1—N2—C17	−92.8 (2)
C12—C13—C14—C15	179.6 (4)	O1^i^—Cd1—N2—C17	87.2 (2)
C13—C14—C15—C16	−0.3 (6)	C3—C2—N3—N4	0.6 (4)
C14—C15—C16—N2	−0.5 (6)	C1—C2—N3—N4	−179.6 (4)
C14—C13—C17—N2	−0.5 (5)	C2—N3—N4—N5	−0.6 (4)
C12—C13—C17—N2	−179.4 (3)	N3—N4—N5—C3	0.4 (4)
C14—C13—C17—C18	179.2 (3)	C2—C3—N5—N4	0.0 (4)
C12—C13—C17—C18	0.3 (5)	C4—C3—N5—N4	−178.4 (4)
C9—C10—C18—N1	−0.1 (6)	N2^i^—Cd1—O1—S1	−34.20 (17)
C11—C10—C18—N1	179.8 (3)	N2—Cd1—O1—S1	145.80 (17)
C9—C10—C18—C17	−179.7 (4)	N1—Cd1—O1—S1	73.52 (17)
C11—C10—C18—C17	0.2 (6)	N1^i^—Cd1—O1—S1	−106.48 (17)
N2—C17—C18—N1	−0.5 (5)	O1^i^—Cd1—O1—S1	−113 (100)
C13—C17—C18—N1	179.8 (3)	Cd1—O1—S1—O3	−158.95 (16)
N2—C17—C18—C10	179.1 (3)	Cd1—O1—S1—O2	−28.3 (2)
C13—C17—C18—C10	−0.6 (5)	Cd1—O1—S1—C1	86.59 (18)
C8—C7—N1—C18	0.4 (6)	C6—C1—S1—O3	99.8 (3)
C8—C7—N1—Cd1	−177.5 (3)	C2—C1—S1—O3	−79.9 (3)
C10—C18—N1—C7	−0.3 (5)	C6—C1—S1—O2	−23.9 (3)
C17—C18—N1—C7	179.3 (3)	C2—C1—S1—O2	156.4 (3)
C10—C18—N1—Cd1	177.8 (3)	C6—C1—S1—O1	−143.5 (3)
C17—C18—N1—Cd1	−2.6 (4)	C2—C1—S1—O1	36.8 (3)
N2^i^—Cd1—N1—C7	1.0 (3)		

Symmetry codes: (i) −*x*, −*y*, −*z*+2.

### Hydrogen-bond geometry (Å, °)

**Table:**

*D*—H···*A*	*D*—H	H···*A*	*D*···*A*	*D*—H···*A*
O1W—H1WA···N5^ii^	0.98	2.10	3.015 (4)	155.
O1W—H1W···O3^iii^	0.98	2.00	2.934 (5)	158.
N3—H3N···O1^iv^	0.90	2.21	3.009 (4)	148.
C6—H6···O2	0.93	2.60	2.947 (4)	103.
C8—H8···N4^v^	0.93	2.42	3.306 (6)	159.
C14—H14···O1W	0.93	2.51	3.414 (5)	164.

Symmetry codes: (ii) *x*, *y*+1, *z*−1; (iii) *x*, *y*, *z*−1; (iv) −*x*+1, −*y*, −*z*+2; (v) *x*−1, *y*+1, *z*.
